# Integrative bioinformatic analysis prioritizes TIMP1 and FN1 as angiogenesis-related candidate genes in diabetic foot ulcers

**DOI:** 10.1371/journal.pone.0348808

**Published:** 2026-05-13

**Authors:** Xiangjun Hu, Degang Dong, Yunfei Wang, Hong Cai, Hongwei Yin, Zhangren Yan

**Affiliations:** 1 Graduate School, Jiangxi University of Chinese Medicine, Nanchang, Jiangxi, China; 2 School of Traditional Chinese Medicine, Jiangxi University of Chinese Medicine, Nanchang, Jiangxi, China; 3 Jiangxi Hospital Affiliated Longhua Hospital, Shanghai University of Traditional Chinese Medicine, Shanghai, China; 4 Department of Traditional Chinese Medicine Surgery, The Affiliated Hospital of Jiangxi University of Chinese Medicine, Nanchang, Jiangxi, China; University of South Carolina School of Medicine, UNITED STATES OF AMERICA

## Abstract

**Background:**

Diabetic foot ulcers (DFUs) represent a severe chronic complication of diabetes and are characterized by persistent impairment of wound healing, accompanied by defective angiogenesis, chronic inflammation, and dysregulated extracellular matrix remodeling. Although impaired angiogenesis is widely recognized as a key pathological feature of DFUs, its associated molecular alterations have not been systematically characterized at the transcriptomic and cellular levels.

**Methods:**

In this study, bulk transcriptomic data were analyzed in combination with machine learning–based gene prioritization and single-cell RNA sequencing to investigate molecular features associated with angiogenesis impairment in DFUs. Differential expression analysis was performed using the GSE199939 and GSE134431 datasets, followed by GO and KEGG enrichment analyses. Angiogenesis-related genes were retrieved from the MSigDB HALLMARK_ANGIOGENESIS and GO:0001525, and intersected with the DEGs to generate a candidate gene set. A LASSO logistic regression model was then constructed in the discovery cohort and evaluated in a replication cohort, yielding a five-gene signature consisting of APLN, ENG, FN1, SERPINA5, and TIMP1. Single-cell transcriptomic data were subsequently used to examine the cellular expression patterns of these feature genes.

**Results:**

Among the five feature genes, FN1 and TIMP1 showed relatively clear expression localization at the single-cell level. Single-cell RNA sequencing analysis revealed that FN1 was mainly enriched in fibroblasts and stromal-related cell populations, including pericytes/smooth muscle cells, whereas TIMP1 exhibited a multicellular expression pattern, with relatively high expression in fibroblasts, inflammatory myeloid cells, macrophages, and proliferating cells. In vivo experiments further showed that TIMP1 and EGFR mRNA expression levels were significantly decreased, whereas MMP9 mRNA expression was significantly increased in wound tissues from the model group. FN1 mRNA showed a downward trend, although the difference did not reach statistical significance.

**Conclusion:**

This integrative bioinformatic analysis provides an exploratory characterization of molecular features potentially related to restricted angiogenesis and impaired repair in DFUs and suggests that TIMP1 may represent a more robust candidate linked to proteolysis-related dysregulation, whereas FN1 may more likely reflect stromal extracellular matrix remodeling.

## 1 Introduction

Diabetic foot ulcers (DFUs) are severe complications arising from chronic inflammation, vascular dysfunction, neuropathy, and immune dysregulation. Hyperglycemia-induced vascular damage creates sustained ischemia that impairs wound repair. DFUs affect 19–34% of diabetic patients, with 65% recurrence, 20% amputation rates, and 70% five-year mortality, reflecting systemic metabolic dysfunction [1,2].

Among the pathological processes involved in DFU progression, impaired angiogenesis is a major driver of delayed healing. Angiogenesis is essential for oxygen and nutrient delivery and for coordinated tissue repair [3]. In the diabetic wound microenvironment, sustained hyperglycemia, oxidative stress, and chronic inflammation impair endothelial function and neovascularization, thereby contributing to restricted vascular regeneration and persistent tissue hypoxia [4]. Although vascular endothelial growth factor (VEGF), hypoxia-inducible factor-1α (HIF-1α), and the PI3K/Akt signaling are known regulators of angiogenesis [5,6], most studies have focused on individual molecules or isolated pathways. A broader transcriptomic view of angiogenesis-related molecular alterations in DFUs remains limited.

With the development of high-throughput omics, computational approaches have become useful for prioritizing candidate genes in complex transcriptomic datasets [7]. When analysis is restricted to a predefined candidate gene set, LASSO logistic regression can support sparse feature selection and model interpretability in small-sample settings. In parallel, single-cell RNA sequencing provides cell-resolution information that helps interpret bulk transcriptomic signals in their cellular context. Based on these considerations, we integrated public DFU transcriptomic datasets and applied LASSO-based candidate prioritization within a predefined angiogenesis-related gene set, followed by single-cell localization analysis. Through this exploratory bioinformatic framework, we aimed to identify angiogenesis-related candidate genes in DFUs and to provide a basis for subsequent functional validation.

## 2 Materials and methods

### 2.1 Data acquisition and preprocessing

The bulk transcriptomic dataset GSE199939 was obtained from the Gene Expression Omnibus (GEO) database and included 10 diabetic wound (DW) samples and 11 normal skin (N) samples [8]. Gene-level expression data were extracted as transcripts per million (TPM) values, with gene symbols used as row identifiers. To reduce the influence of low-expression noise, low-expression filtering was performed: genes were retained for downstream analyses if they showed TPM > 1 in at least 3 samples in either the DW group or the N group. The expression matrix was then log₂-transformed using log₂(TPM + 1) to stabilize variance and improve data distribution, yielding the final matrix for downstream analyses.

In addition, the GSE134431 dataset was downloaded as a replication cohort, consisting of 13 non-healing diabetic foot ulcer (DFU) samples and 8 diabetic foot skin (DFS) samples [9]. The expression matrix of this cohort was provided in RPKM format and was transformed using log₂(RPKM+1) before construction of the replication cohort expression matrix. Given the differences between the two datasets in platform, quantification unit, and comparison groups, GSE199939 was defined as the discovery cohort and GSE134431 as the replication cohort; the two datasets were analyzed independently and were not merged for joint analyses. The processed log2-transformed expression matrices used for the discovery and replication cohorts are provided in S1 and S2 Datas, respectively.

### 2.2 Differential expression analysis

Differential expression analysis was conducted on the GSE199939 dataset using the limma package (version 3.64.3) in R (version 4.5.1). Linear models combined with empirical Bayes moderation were applied to assess gene expression differences between diabetic wound and normal skin samples. P-values were adjusted for multiple testing using the Benjamini-Hochberg (BH) method. Genes with an adjusted P value (adj. P value) < 0.05 and an absolute log₂ fold change (|log₂FC|) > 1.5 were defined as DEGs. The differential expression results were visualized using volcano plots and heatmaps.

### 2.3 Functional enrichment analysis

To investigate the potential biological functions of DEGs, Gene Ontology (GO) functional enrichment analysis and Kyoto Encyclopedia of Genes and Genomes (KEGG) pathway enrichment analysis were performed using the clusterProfiler package (version 4.16.0) in R. All detected genes were used as the background gene set. Enriched terms with an adj. P value < 0.05 were considered statistically significant.

### 2.4 Angiogenesis-related gene set acquisition and integration

To identify genes associated with angiogenesis for subsequent analyses, the HALLMARK_ANGIOGENESIS gene set was retrieved from the Molecular Signatures Database (MSigDB). In addition, angiogenesis-related genes were obtained from the Gene Ontology biological process term GO:0001525 (angiogenesis). The two gene sets were integrated, and duplicate genes were removed to generate a consolidated angiogenesis-related gene set (S1 Table). This gene set was then intersected with the differentially expressed genes (DEGs) identified in the discovery cohort to obtain angiogenesis-related DEGs, which were subsequently used for machine learning-based feature selection and subsequent evaluation.

### 2.5 Machine learning-based candidate gene prioritization

Based on the angiogenesis-related DEGs, LASSO logistic regression was applied for feature selection. GSE199939 was used as the discovery cohort and GSE134431 as the replication cohort. Expression matrices from both cohorts were aligned to the candidate gene set, and only shared genes were retained for model construction. Features were standardized using z-scores, and the scaling parameters derived from the discovery cohort were applied to the replication cohort. Given the limited sample size of the discovery cohort, no additional internal random data splitting was performed. Accordingly, the LASSO analysis was intended for candidate prioritization rather than for developing a clinically applicable predictive model. Instead, stratified five-fold cross-validation was used to determine the regularization parameter λ. A 9-gene model was extracted at λ.1se. To improve model parsimony and interpretability in this small-sample setting, candidate solutions with no more than five non-zero coefficients along the LASSO path were examined, and the solution with the lowest cross-validation error was selected to construct the 5-gene signature as the main model. Model fitting was performed in the discovery cohort, and discrimination performance was primarily assessed in the replication cohort using receiver operating characteristic (ROC) curves and the area under the ROC curve (AUC) with 95% confidence intervals. In the replication cohort, the distribution of predicted probabilities was compared using the Wilcoxon rank-sum test, and the AUCs of the 9-gene and 5-gene models were compared using DeLong’s test. Because the comparison framework differed between the two cohorts (DW vs. N in the discovery cohort and DFU vs. DFS in the replication cohort), GSE134431 was treated as an independent replication assessment rather than a strict external validation.

### 2.6 Single cell transcriptomic analysis and gene localization

To examine the cellular distribution of selected genes in diabetic wound tissues, the single-cell RNA sequencing dataset GSE165816 was downloaded and analyzed [10]. Raw count matrices from each sample were imported to construct Seurat objects, which were then merged for downstream analyses. Quality control (QC) was performed to remove low-quality cells according to the following criteria: nFeature_RNA < 200 or > 6000, and mitochondrial gene proportion > 15%. The remaining cells were normalized using the LogNormalize method, and the top 2000 highly variable genes were identified. ScaleData and principal component analysis (PCA) were then performed, and the first 15 principal components were selected based on the elbow plot for neighborhood detection (FindNeighbors, k = 30), clustering (FindClusters, resolution = 0.2), and Uniform Manifold Approximation and Projection (UMAP) visualization, yielding 14 cell clusters.

Cluster-specific marker genes were identified using the FindAllMarkers. To reduce computational burden, marker identification was performed in a randomly downsampled subset of 5000 cells. The expression patterns of key genes across different cell types were visualized using DotPlot, FeaturePlot, VlnPlot, and heatmaps. Expression differences among cell types were further assessed using Kruskal–Wallis and pairwise Wilcoxon rank-sum tests with Benjamini–Hochberg correction. The analysis scripts for the bioinformatic analyses described above are provided in S1 Text.

### 2.7 Animal model construction, histological evaluation, and assessment of Key mRNA expression

All animal procedures were approved by the Animal Ethics Committee of Jiangxi University of Chinese Medicine (Approval No. JZLLSC20260652) and conducted in accordance with institutional guidelines. A diabetic rat model was established using a high-fat diet combined with streptozotocin (STZ) injection as previously described [11], and successful model establishment was confirmed according to the referenced protocol. After successful modeling, a 6 × 6 mm full-thickness excisional wound was created on the dorsal skin.

On day 14, wound and peri-wound tissues were collected. A portion of each sample was fixed in 4% paraformaldehyde for 24 h, paraffin-embedded, and sectioned at 4 μm for H&E and Masson’s trichrome staining. The remaining tissue was snap-frozen in liquid nitrogen and stored at −80 °C for RT-qPCR. Total RNA was extracted using Genestar reagents and reverse-transcribed into cDNA. RT-qPCR was performed to quantify FN1, TIMP1, MMP9, and EGFR, using GAPDH as the internal reference. Primer sequences are listed in S2 Table. Four samples per group were analyzed, and relative expression levels were calculated using the 2^-ΔΔCt method. Technical replicates were included for each sample. Data are presented as mean ± SD. Normally distributed data were analyzed using an unpaired Student’s t-test, and non-normally distributed data were analyzed using the Mann–Whitney U test. P < 0.05 was considered statistically significant.

## 3 Results

### 3.1 Identification of differentially expressed genes

**Fig 1:**
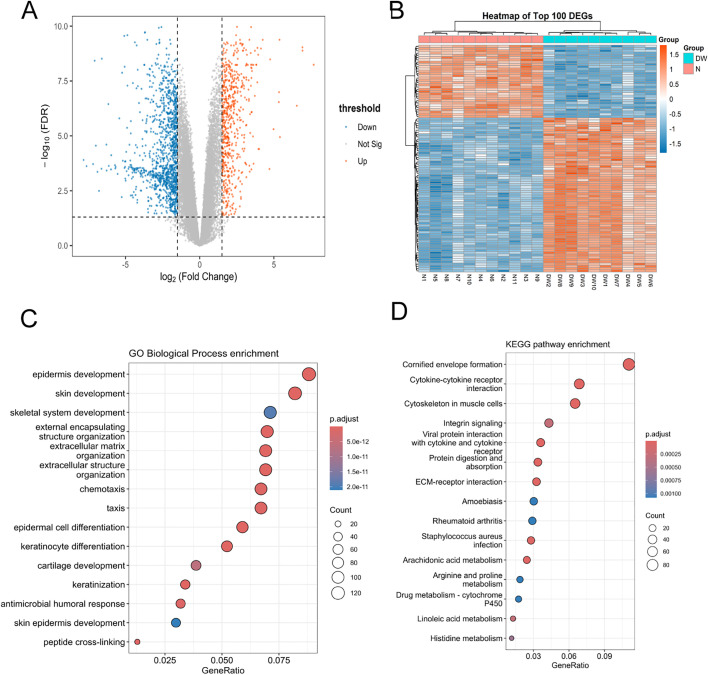
Differential expression and functional enrichment analyses in the discovery cohort. **(A)** Volcano plot of DEGs between DW and normal skin N samples. Red and blue dots indicate upregulated and downregulated genes, respectively. **(B)** Heatmap of the top 100 DEGs showing expression patterns across DW and N samples. Red and blue indicate relatively high and low expression, respectively. **(C)** GO biological process enrichment analysis of DEGs. **(D)** KEGG pathway enrichment analysis of DEGs.7.

Differential expression analysis was performed on the GSE199939 dataset. A total of 39,956 genes were detected, among which 1,802 genes met the predefined criteria for differential expression (adj. P value < 0.05 and |log₂ FC| > 1.5; S3 Table). Of these, 594 genes were upregulated and 1,208 genes were downregulated in diabetic wound tissues (Fig 1A). The volcano plot showed a clear distribution of upregulated and downregulated genes. Hierarchical clustering of the top 100 DEGs ranked by adjusted P value further separated DW and N samples, with good within-group consistency (Fig 1B).

Among the upregulated genes, several genes related to cell proliferation and mitosis, including MYBL2, AURKA, CCNB1, CDC20, and MKI67, showed markedly increased expression. In addition, several genes involved in matrix-vascular regulation, such as APLN, ENG, FN1, ITGAV, ITGB3, PGF, and COL4A1, were also upregulated, indicating altered matrix remodeling- and angiogenesis-related transcriptional programs in diabetic wound tissues. In contrast, downregulated genes included VIPR1, FBLN5, NDRG2, CXCL14, and TIMP3, which are associated with skin homeostasis, matrix maintenance, and tissue repair.

### 3.2 Functional enrichment analysis of DEGs

To examine the functional characteristics of the identified DEGs, GO and KEGG enrichment analyses were performed (Fig 1C–1D; S4–S5 Tables). GO biological process enrichment showed that DEGs were mainly associated with skin development and epidermis-related programs, including epidermis development, keratinocyte differentiation, keratinization, and epidermal cell differentiation, as well as extracellular matrix organization-related terms. In addition, chemotaxis/taxis-related terms were also significantly enriched, suggesting persistent activation of immune cell recruitment and inflammatory migration in diabetic wound tissues (Fig 1C; S4 Table).

KEGG pathway enrichment identified several significantly enriched pathways, including cornified envelope formation (hsa04382), cytokine–cytokine receptor interaction (hsa04060), ECM–receptor interaction (hsa04512), and multiple metabolism-related pathways, including arachidonic acid metabolism (hsa00590), linoleic acid metabolism (hsa00591), and histidine/arginine and proline metabolism (Fig 1D; S5 Table). Overall, the enrichment results indicated that aberrant repair in diabetic wound tissues was mainly characterized by dysregulation of epidermal differentiation and barrier reconstruction, inflammatory chemotaxis and cytokine signaling, extracellular matrix remodeling, and metabolic reprogramming.

### 3.3 Machine learning prioritized angiogenesis-related candidate genes in diabetic wounds

**Fig 2:**
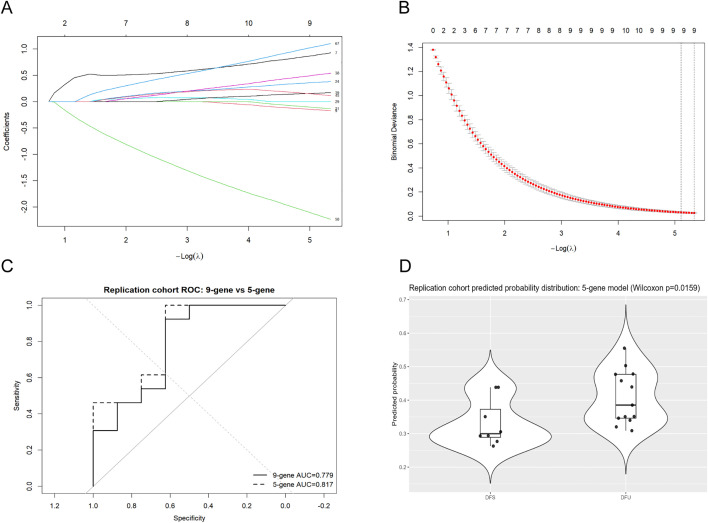
Construction and evaluation of angiogenesis-related LASSO gene signatures in the discovery and replication cohorts. **(A)** LASSO coefficient profiles of candidate genes in the discovery cohort. **(B)** Five-fold cross-validation curve for selection of the optimal penalty parameter in the discovery cohort. **(C)** ROC curves of the 9-gene and 5-gene models in the replication cohort (DFS vs. DFU). Solid and dashed lines indicate the 9-gene and 5-gene models, respectively. **(D)** Predicted probability distribution of the 5-gene model in the replication cohort. Each dot represents one sample, and group differences were compared using the Wilcoxon test.

A total of 73 angiogenesis-related candidate genes were identified in the discovery cohort after intersection analysis (S6 Table). After feature alignment between the discovery cohort (GSE199939) and the replication cohort (GSE134431), 72 shared genes were retained for model construction because one gene was unavailable in the replication cohort. Using the z-score-standardized expression matrix of the discovery cohort, a LASSO logistic regression model was established with stratified five-fold cross-validation. As the penalty parameter λ decreased, the number of genes with non-zero coefficients increased, whereas the binomial deviance decreased and then stabilized (Fig 2A, 2B). Under the λ.1se criterion, a 9-gene feature set was obtained, including APLN, APOD, CX3CL1, ENG, ITGAV, ITGB3, MYDGF, SERPINA5, and TIMP1 (S7 Table). Among candidate solutions with no more than five non-zero coefficients, the one with the lowest cross-validation error was selected, yielding a 5-gene signature consisting of APLN, ENG, FN1, SERPINA5, and TIMP1 (S7 Table). The coefficients and absolute coefficient ranking of all candidate genes under the λ.1se criterion are provided in S8 Table.

In the replication cohort, the 5-gene model showed a numerically higher AUC than the 9-gene model (0.817 vs. 0.779) (Fig 2C; S9 Table). However, the difference between the two ROC curves was not statistically significant according to DeLong’s test (P = 0.146; 95% CI for the AUC difference, −0.090 to 0.013). In addition, the predicted probabilities generated by the 5-gene model differed significantly between the two groups (Wilcoxon rank-sum test, P = 0.0159; Fig 2D; S10 Table). Taken together, these findings suggest that the parsimonious 5-gene model retained discriminatory ability comparable to that of the 9-gene model in a second small-sample cohort. These results provide supportive replication evidence, but should not be interpreted as strict external validation.

### 3.4 Single-cell transcriptomic analysis of diabetic wound tissues

**Fig 3:**
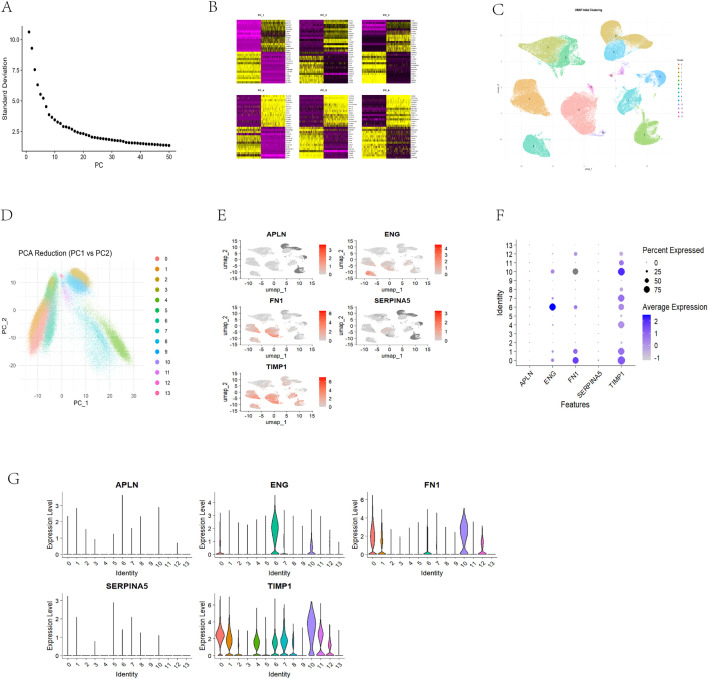
Single-cell transcriptomic profiling of diabetic wound tissue and expression mapping of the 5-gene signature. **(A)** Elbow plot supporting the selection of the top 15 principal components for downstream analyses. **(B)** Heatmaps of the top gene loadings for PC1–PC6. **(C)** UMAP projection of 14 unsupervised clusters identified in GSE165816. **(D)** PCA projection (PC1 vs. PC2) showing the distribution of the identified clusters. **(E)** Feature plots showing the single-cell expression distribution of APLN, ENG, FN1, SERPINA5, and TIMP1. **(F)** Dot plot summarizing the expression patterns of the 5 genes across clusters. Dot size indicates the percentage of expressing cells, and dot color indicates the average expression level. **(G)** Violin plots showing the expression distributions of the 5 genes across clusters.

To characterize the cellular composition of diabetic wound tissues and evaluate the expression localization of candidate feature genes at single-cell resolution, the GSE165816 dataset was analyzed. After merging the raw count matrices, 174,962 cells were obtained, of which 162,206 high-quality cells were retained after QC. After normalization and highly variable gene selection, PCA was performed, and the first 15 principal components were selected for clustering based on the elbow plot (Fig 3A). Gene loadings across PC1–PC6 showed clear differences (Fig 3B). Unsupervised clustering based on the first 15 principal components at a resolution of 0.2 identified 14 transcriptionally distinct cell clusters (Fig 3C). Both UMAP and PCA projections showed clear separation among clusters (Fig 3C, 3D), indicating marked cellular heterogeneity in diabetic wound tissues. Marker genes and cell counts for the identified clusters are summarized in S11 Table.

The five feature genes, APLN, ENG, FN1, SERPINA5, and TIMP1, were then mapped onto the single-cell atlas. Their expression localization differed markedly across cells (Fig 3E). APLN, ENG, and SERPINA5 showed generally weak expression and low proportions of positive cells, whereas FN1 and TIMP1 exhibited clearer signals and were detected in a higher proportion of cells (Fig 3F, 3G). FN1 and TIMP1 were therefore selected for subsequent cell-type localization analysis.

### 3.5 Cell type annotation and single-cell localization of FN1 and TIMP1

**Fig 4:**
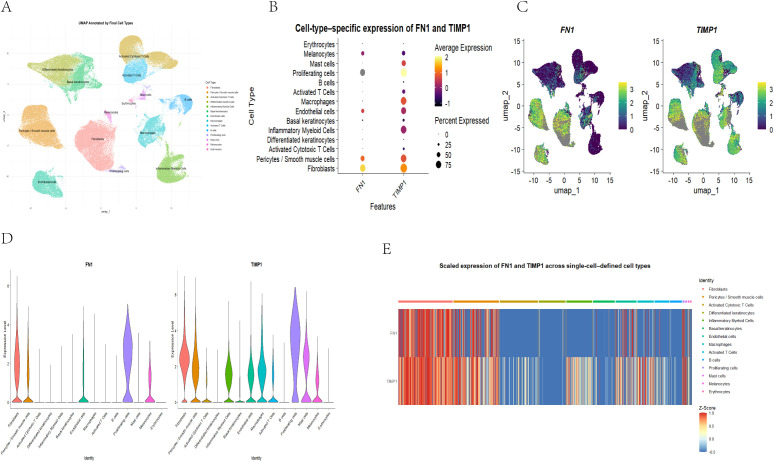
Cell-type annotation and single-cell localization of FN1 and TIMP1 in diabetic wound tissue. **(A)** UMAP visualization of annotated cell populations in the GSE165816 single-cell dataset, identifying 14 major cell types. **(B)** Dot plot showing the expression patterns of FN1 and TIMP1 across annotated cell types. Dot size indicates the percentage of expressing cells, and dot color indicates the average expression level. **(C)** Feature plots showing the expression distributions of FN1 and TIMP1 on the UMAP projection. **(D)** Violin plots showing the expression distributions of FN1 and TIMP1 across annotated cell types. **(E)** Heatmap summarizing the scaled expression patterns of FN1 and TIMP1 across annotated cell types.

Based on the unsupervised clustering results, cell type annotation was performed by integrating canonical marker genes with cluster-specific expression patterns. A total of 14 major cell populations were identified, including Fibroblasts, Pericytes/Smooth muscle cells, Activated Cytotoxic T cells, Differentiated keratinocytes, Inflammatory Myeloid Cells, Basal keratinocytes, Endothelial cells, Macrophages, Activated T cells, B cells, Proliferating cells, Mast cells, Melanocytes and Erythrocytes (Fig 4A).

The single-cell expression patterns of FN1 and TIMP1 were then examined across the annotated cell types. Dot plot analysis showed that FN1 was mainly expressed in Fibroblasts and Pericytes/Smooth muscle cells, with additional signals in Proliferating cells. In contrast, TIMP1 showed a broader expression pattern, with relatively high expression in Fibroblasts, Pericytes/Smooth muscle cells, Inflammatory Myeloid Cells, Macrophages, Proliferating cells, and some Mast cells (Fig 4B). Feature plots, violin plots, and the standardized heatmap further supported these findings, showing that FN1 exhibited a stromal-associated expression pattern, whereas TIMP1 showed a broader cross-cell-type distribution (Fig 4C–4E). Statistical comparisons of FN1 and TIMP1 expression across annotated cell types are provided in S12 Table.

Overall, FN1 was mainly enriched in stromal and perivascular-related cell populations, whereas TIMP1 was distributed across both stromal and immune/inflammation-related cell populations, suggesting differences in their cellular sources and potential functional scope within the diabetic wound microenvironment.

### 3.6 In vivo wound phenotype, histology, and candidate gene mRNA expression

**Fig 5:**
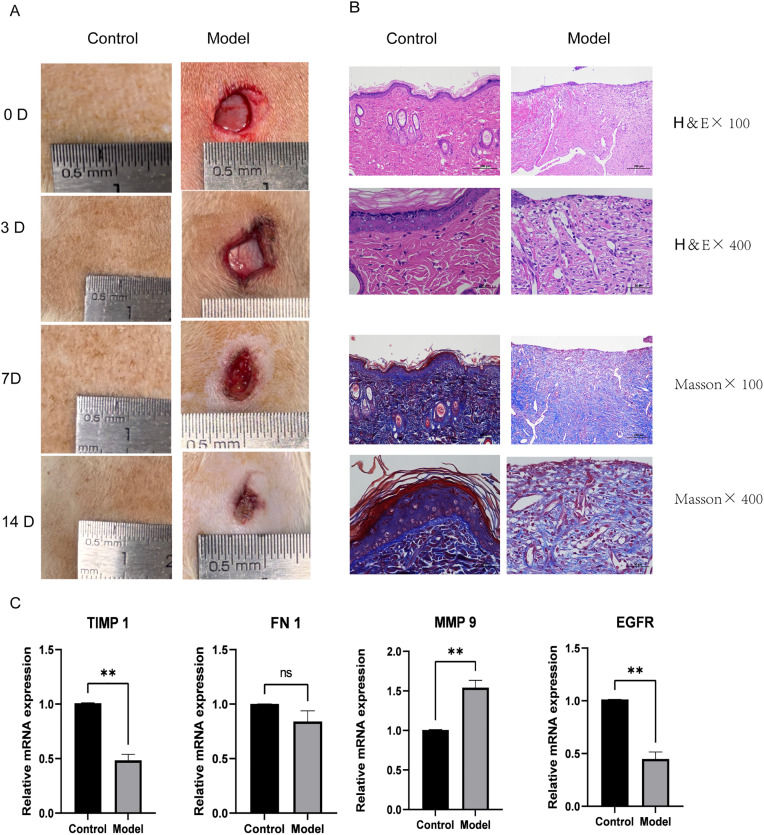
Gross wound appearance, histological changes, and mRNA expression of selected genes in the animal wound model. **(A)** Representative gross images of the control and model groups at 0, 3, 7, and 14 days after wound creation. **(B)** Representative H&E and Masson staining images of wound tissues from the control and model groups at day 14, shown at H&E × 100, H&E × 400, Masson ×100, and Masson ×400. **(C)** RT-qPCR analysis of TIMP1, FN1, MMP9, and EGFR mRNA expression in wound tissues at day 14. ns, not significant; *P < 0.05; **P < 0.01.

In the model group, a 6 × 6 mm full-thickness excisional wound was created on the dorsal skin, and wound photographs were obtained on days 0, 3, 7, and 14 after wound creation. Visible wounds and dynamic healing changes were observed in the model group at all time points, whereas no wound formation was observed at the corresponding skin site in the control group, which remained grossly intact (Fig 5A). By day 14, the wound area in the model group had decreased compared with earlier time points, but residual wound tissue and incomplete healing were still evident.

Histological examination on day 14 showed that the skin structure in the control group remained intact, whereas the model group exhibited local tissue disorganization, incomplete epidermal repair, and abnormal collagen deposition and arrangement, indicating that the wound remained in a state of aberrant repair (Fig 5B). Further RT-qPCR analysis showed that, compared with the control group, TIMP1 and EGFR mRNA expression levels were significantly decreased in the model group, whereas MMP9 mRNA expression was significantly increased. FN1 mRNA showed only a downward trend, without reaching statistical significance (Fig 5C; S13 Table).

## 4 Discussion

By integrating multi-cohort transcriptomics, machine learning, and single-cell localization analysis, this study provides an exploratory view of angiogenesis-associated dysregulation in DFUs. Rather than attributing defective vascular repair solely to endothelial abnormalities, our findings suggest that altered FN1 and TIMP1 expression reflects disequilibrium among stromal remodeling, persistent inflammation, and restricted vascular repair in the chronic DFU microenvironment [12,13]. In human public datasets, both genes showed an overall upward trend, suggesting ongoing matrix remodeling and protease network regulation in DFU tissues. However, this upregulation does not necessarily indicate effective angiogenesis or tissue maturation, but may instead reflect sustained stress-responsive and compensatory remodeling under chronic injury.

Notably, despite this overall upregulation in human public datasets, RT-qPCR assessment on day 14 in the diabetic wound model, using mixed wound bed and peri-wound tissues, showed significant downregulation of TIMP1, whereas FN1 showed only a downward trend without statistical significance. Because both analyses used normal skin as the reference, this discrepancy suggests that FN1 and TIMP1 expression may be influenced by healing stage, sampling context, and tissue source, as well as by species differences. Accordingly, these changes may better reflect extracellular matrix (ECM) turnover and inflammatory regulation than angiogenic activity itself.

To further interpret this discrepancy, we additionally examined MMP9 and EGFR, two molecules related to key pathological processes in chronic wounds. In the model group, MMP9 was significantly increased, whereas TIMP1 and EGFR were significantly decreased. This pattern suggests that the day-14 wound remained in a restricted repair state with increased proteolytic pressure and insufficient re-epithelialization drive. Specifically, increased MMP9 together with reduced TIMP1 suggests imbalance in the protease–inhibitor axis, which may be unfavorable for stable ECM turnover, whereas reduced EGFR is consistent with suppressed epithelial proliferation and re-epithelialization signaling and with delayed wound closure. Importantly, MMP9 and EGFR were not intended as direct markers of angiogenesis, but as supportive indicators of increased proteolytic burden and insufficient epithelial repair. These findings suggest temporal non-synchrony among ECM remodeling, proteolytic stress, and epithelial repair.

TIMP1 is an important regulator of MMP activity and ECM turnover balance [14,15]. In the present multi-cohort transcriptomic analysis, TIMP1 showed an overall upregulated trend, whereas single-cell analysis indicated a multicellular expression pattern, particularly in myeloid cells, macrophages, and proliferating cells. Together with the in vivo findings of reduced TIMP1 and increased MMP9, these results suggest that TIMP1 alterations in DFUs may reflect proteolysis-related remodeling under chronic inflammation rather than effective repair. This interpretation is supported by animal studies [16] and by human evidence showing that enhanced protease-related signals in DFU wound exudates are associated with poor healing, while inclusion of inhibitors such as TIMP-1 may improve discrimination of healing outcomes [17]. Previous studies further suggest that the role of TIMP1 in diabetic chronic wounds may vary with wound-healing stage, local microenvironment, and dominant expressing cell populations [18,19]. Therefore, the biological significance of TIMP1 in DFUs may be better understood within the pathological context of persistent inflammation, abnormal proteolytic load, and ECM turnover imbalance. Further studies integrating MMP activity profiles, ECM structural phenotypes, and vascular maturation-related indicators are needed to clarify its role across different stages of repair.

FN1 is an important adhesive ECM component in wound repair. It participates in fibronectin fibril assembly and regulates cell adhesion, migration, and tissue remodeling through integrin-related pathways [20,21]. Under chronic hyperglycemic and inflammatory conditions, the biological significance of FN1 may depend more on its effective deposition and ordered assembly within the wound bed than on transcript abundance alone. Previous studies have shown that elevated protease activity in DFU wounds may disrupt fibronectin deposition and fibril assembly, thereby weakening the matrix support required for cell migration and tissue repair [22]. FN1 may also participate in pro-repair responses under specific signaling contexts, although its expression changes do not necessarily parallel vascular reconstruction [23]. In our single-cell analysis, FN1 was mainly enriched in stromal-related populations, including fibroblasts and pericytes/smooth muscle cells, consistent with a role in stromal ECM remodeling rather than angiogenic activity alone [24]. In vivo, FN1 showed only a nonsignificant downward trend, which may reflect stage-related and sampling-related variation. Overall, FN1 may be better regarded as a candidate marker of stromal ECM remodeling in DFUs, with its association with restricted angiogenesis likely mediated indirectly through abnormal matrix scaffold remodeling.

Overall, this study suggests that TIMP1 may be a more robust candidate gene linked to proteolysis-related dysregulation and restricted repair in DFUs, whereas FN1 may better reflect stromal ECM assembly and remodeling. Together, these two molecules provide clues to the molecular basis of restricted vascular repair in diabetic wounds.

Several limitations should be acknowledged. Public transcriptomic datasets differed in sample source, sequencing platform, and preprocessing workflow, so cross-cohort heterogeneity and residual batch effects cannot be fully excluded despite separate within-cohort preprocessing and aligned downstream analyses. In addition, the overall sample size was limited, and the replication cohort was small and not fully phenotypically matched to the discovery cohort; therefore, the findings should be interpreted as replication support rather than strict external validation. Machine learning–based screening in a small-sample setting may also suffer from unstable feature selection and limited generalizability; accordingly, the present model is better suited to candidate prioritization and hypothesis generation than to clinical prediction. Finally, transcript-level changes do not necessarily reflect protein abundance, spatial deposition, or enzymatic activity, and further validation with proteomics, spatial omics, quantitative histology, and in vitro/in vivo functional experiments is required.

Despite these limitations, this study provides new clues to the molecular background linking disturbed matrix remodeling with restricted repair in DFUs and highlights the proteolysis-related network represented by TIMP1 and the stromal ECM program represented by FN1 as areas warranting further investigation. Future work should validate these findings in larger, multicenter cohorts and integrate temporal, spatial, and functional analyses to clarify the specific roles of these two molecules in aberrant diabetic wound repair.

## 5 Conclusions

Overall, this study prioritizes TIMP1 and FN1 as exploratory candidate genes linked to proteolysis-related dysregulation and stromal ECM remodeling, respectively, in DFUs. Their potential relevance to restricted angiogenesis and impaired repair warrants further functional investigation.

## Supporting information

S1 Table Consolidated angiogenesis-related gene set. (TXT)

S2 Table Primer sequences used for RT-qPCR. (XLSX)

S3 Table Complete differential expression results. (CSV)

S4 Table GO biological process enrichment results of DEGs. (CSV)

S5 Table KEGG pathway enrichment results of DEGs. (CSV)

S6 File Core_intersection. (TXT)

S7 Table 9-gene and 5-gene LASSO coefficients. (XLSX)

S8 Table LASSO coefficients and rankings at lambda.1se. (CSV)

S9 Table 9-gene and 5-gene model performance. (XLS)

S10 Table 9-gene and 5-gene predicted probabilities. (XLSX)

S11 Table scRNA-seq cluster markers and cell counts. (XLS)

S12 Table scRNA_summary. (XLSX)

S13 Table RT-qPCR Ct and normalized expression data. (XLSX)

S1 Data GSE199939 processed log2 expression matrix. (TXT)

S2 Data GSE134431 processed log2 expression matrix. (TXT)

S1 Text Code. Analysis scripts. (TXT)
